# Case of Gastrotomy for the Removal of a Leaden Bar

**Published:** 1855-05

**Authors:** T. B. Neal

**Affiliations:** Columbus City, Iowa


### SELECTIONS.

From the Medical Examiner.

*Case of Gastrotomy for the Removal of a Leaden Bar; Recovery.* By T. B. Neal, M.D., Columbus City, Iowa.

Editor Medical Examiner:

Dear Sir—I transmit, for insertion in your valuable journal, the following remarkable and perhaps unique case:

The subject of this notice, L. Bates, æt. 27, resides at Wapello, twelve miles from this city. During the three days preceding Christmas last he had been drinking of common whiskey; and on that day, while intoxicated, attempted, on a wager, to swallow a bar of lead. The bar was 10 inches long, ½ by ¾ inches thick, and weighed one pound

Thrusting it far down the œsophagus, it slipped from his grasp, and immediately entered his stomach. Dr. Bell was sent for at once, but as Bates had formerly been a juggler, the Doctor, thinking that he was at some of his tricks, refused to go. Bates, not much concerned at the non-attendance of the physician, worked for three days after the accident in a pork-house, with but little inconvenience. During the night of the third day, however, he was seized with great pain in the stomach, accompanied with shooting pains along the spine, extending from the lumbar region to the sacrum, and thence to the hips. The next day he walked to Columbus, a distance of six miles, and sent for Dr. Robertson, the oldest physician in this county, to attend upon him. Dr. R.

requested me to see the case with him. We found him, on the fourth day, comparatively easy. His tongue was white, breath very foul, and bowels constipated. Upon careful examination the œsophagus was found perfectly free and unobstructed. We administered to him morphia in small doses, and attempted to act upon his bowels, and neutralize the poisonous effects of the lead by large doses of sulphate of magnesia. Under this treatment, although the bowels were but slightly disturbed, he was rendered astonishingly comfortable, and could walk about a little. On the 3d of January, the tenth day after the accident, the severe gastric pain again returned, accompanied with vomiting, and other symptoms of gastritis.

The operation of gastrotomy was now resolved upon. Dr. Bell, of Wapello, performed the operation by making an incision through the walls of the abdomen, from the umbilicus to the false ribs, four inches in length and two inches to the left of the median line. The peritoneum being divided, Dr. Bell introduced his hand, and pushing back the protruding intestines, found that the bar of lead was nearly perpendicular, the upper end inclining a little to the left. The bar was pushed up, until the lower end came opposite the abdominal opening. It was then seized, and an incision made in the walls of the stomach, just large enough to admit of its extraction by means of forceps. The contraction of the muscular coat of the stomach caused the incision in the organ to close perfectly and without trouble. The external wound was stitched, and a compress applied.

The operation was performed between three and four o'clock P.M., the day was cloudy, and towards sunset grew quite cold. The patient was entirely under the influence of chloroform until about two minutes before the last stitch was taken, when he revived somewhat, and expressed himself as feeling better than he had done before. When the chloroform was first administered to him, he vomited freely; hence, when the opening was made in the stomach, nothing escaped therefrom, that viscus containing nothing but the leaden bar.

For the ensuing three days the system of the patient was kept under the influence of opium, and nothing but mucilaginous drinks, in small quantities, allowed as diet. He recovered as well as a patient does of uncomplicated gastritis.

I give you below the condensed notes of the progress of the case, taken by Drs. Bell, Robertson and myself.

January 4th, 10 A.M.—Patient tolerably quiet; pulse 85, and moderately full; some fever and thirst; vomited once, and bowels moved freely during the night, though he has been taking small doses of morphia every two hours; complains of pain in the stomach and bowels; continued the morphia, and ordered two table-spoonfuls of toast and water every two hours.

3 P.M.– Patient still quiet: pulse 85, and rather hard. Took ℥x. blood from the arm. Continued morphia and toast-water.

January 5th, 10 A.M.—Rested well last night; bowels undisturbed for eight hours; then at 4 A.M. watery dejections; complains of nausea; pulse 83, and soft; tongue white; no soreness in the gastric region; some cough; ordered the following pill every two hours and a half:

| | | |
|---|---|---|
| ℞ | Hydrarg. chlorid. mit. | gr. ss. |
| | Pulv. ipecacuanhæ | gr. j. |
| | Morphiæ sulphat. | gr. ¼. |
| M. ft. pil. i. | | |

5 P.M.—Complains of heart-burn, nausea, thirst, and frequent alvine evacuations; pulse 73, strong and full; craves acid drinks. Prescribed a weak solution of citric acid; it did not appear to agree with the stomach. At 9 o clock slight vomiting: therefore directed morphia' gr. ss., ipecac. pulv. gr. ii. At 11½ o'clock administered morphia, gr. ss. In half an hour patient fell into an easy sleep, his pulse growing softer. Directed gum-water and ice-water, a tablespoonful alternately every half hour, and the morphia every two and a half hours.

January 14th.—Met patient standing in the door; appetite good; pulse 70, and soft; wound looks well; dressed it, and ordered one blue pill, to be followed by enema if it failed to operate.

On the 16th the wound was nearly healed, and he had walked half a mile to see a neighbor. At the time of writing this hasty epistle, (Feb. 19th,) he has been out of the settlement some two weeks, and I learn has been at work. Wdat his exact condition is at present I do not know, not having seen him since he began to go abroad. Yours respectfully, &c.,

T. B. Neal.

---

Note.—There must be very few cases of this operation on record, as we have succeeded in finding but one, which is described in Chelius' System of Surgery, in a note by Mr. South: "Barnes," he says, "quotes from Becker the case of a young peasant, who on the 29th of May, 1635, whilst endeavoring to produce vomiting with the handle of a knife, let it slip from his fingers, and pass into the stomach. He was much frightened, but able to go about his usual occupation. It was, however, determined to remove the knife by operation, which was done on the 9th of July following, by a surgeon and lithotomist, named Shoval 'A straight incision was made in the left *hypoohendrium*, two fingers breadth under the false ribs, first through the skin and cellular membrane, then through the muscles and *peritoneum*. The stomach subsided and slipped from the fingers, which prevented it from being immediately seized; but it was at length caught hold of with a curved needle, and drawn out of the wound. A small incision was then made into it upon the knife, which was then easily extracted. The stomach immediately collapsed. After the external wound had been properly cleansed, it was united with five sutures, and tepid balsam poured into the interstices. Tents impregnated with the same balsam, and a cataplasm of bolar earth, the white of egg, and alum were then applied.' (P. 324.) Two sutures were removed next day; on the following day two more; but the fifth is not not noticed. On the fourteenth day after the operation the wound had healed Dr. Oliver saw this knife at Konigsberg in 1635, and says it was six inches and a half long. The patient completely recovered."

This is the only instance of gastrotomy proper we have been able to find recorded. The rarity of the operation is no doubt owing to the fact that most substances capable of entering the stomach pass eventually, even though quite bulky, into the intestine; in which case enterotomy may be required for their extraction. Of this latter operation, for the relief either of intestinal obstructions or the removal of foreign bodies in the bowels, numerous cases are on record. Mr. Benj. Philips, in an excellent monograph, published in the 31st volume of the Royal Medico Chirurgical Transactions, relates twenty-seven cases of intestinal obstruction, for which operations were performed. Thirteen of these were successful, though many of them were at the cost of establishing an artificial anus. An interesting case is relaied by Dr. White, of New York in the New York Medical Repository, Vol. X., 1807, where a silver spoon, swallowed by a patient while delirious, was removed from the ileum.— EDITOR.

